# Developing a reporting guideline for social and psychological intervention trials

**DOI:** 10.1186/1745-6215-14-242

**Published:** 2013-08-01

**Authors:** Evan Mayo-Wilson, Sean Grant, Sally Hopewell, Geraldine Macdonald, David Moher, Paul Montgomery

**Affiliations:** 1 Centre for Outcomes Research and Effectiveness, Research Department of Clinical, Educational & Health Psychology, University College London, 1-19 Torrington Place, London WC1E 7HB, UK; 2 Centre for Evidence-Based Intervention, University of Oxford Barnett House, 32 Wellington Square, Oxford OX1 2ER, UK; 3 Centre for Statistics in Medicine, University of Oxford Wolfson College Annexe, Linton Road, Oxford OX2 6UD, UK; 4 Institute of Child Care Research, Queen’s University Belfast, 6 College Park, Belfast BT7 1LP, UK; 5 Clinical Epidemiology Program, Ottawa Hospital Research Institute Centre for Practice-Changing Research (CPCR), The Ottawa Hospital − General Campus, 501 Smyth Rd Room L1288, Ottawa, ON K1H 8L6, Canada

**Keywords:** Randomised controlled trial, RCT, CONSORT-SPI, Reporting guideline, Reporting standards

## Abstract

Social and psychological interventions are often complex. Understanding randomised controlled trials (RCTs) of these complex interventions requires a detailed description of the interventions tested and the methods used to evaluate them; however, RCT reports often omit, or inadequately report, this information. Incomplete and inaccurate reporting hinders the optimal use of research, wastes resources, and fails to meet ethical obligations to research participants and consumers. In this paper, we explain how reporting guidelines have improved the quality of reports in medicine, and describe the ongoing development of a new reporting guideline for RCTs: CONSORT-SPI (an Extension for social and psychological interventions). We invite readers to participate in the project by visiting our website, in order to help us reach the best-informed consensus on these guidelines (http://tinyurl.com/CONSORT-study).

## Background

Social and psychological interventions aim to improve physical health, mental health, and associated social outcomes. They are often complex and typically involve multiple, interacting intervention components (for example, several behaviour change techniques) that may act and target outcomes on several levels (for example, individual, family, community) [1]. Moreover, these interventions may be contextually dependent upon the hard-to-control environments in which they are delivered, (for example, health care settings, correctional facilities) [2,3]. The functions and processes of these interventions may be designed to accommodate particular individuals or contexts, taking on different forms while still aiming to achieve the same objective [4,5].

Complex interventions are common in public health, psychology, education, social work, criminology, and related disciplines. For example, multisystemic therapy (MST) is an intensive intervention for juvenile offenders. Based on social ecological and family systems theories, MST providers target a variety of individual, family, school, peer, neighbourhood, and community influences on psychosocial and behavioural problems [6]. Treatment teams of professional therapists and caseworkers work with individuals, their families, and their peer groups to provide tailored services [7]. These services may be delivered in homes, social care, and community settings. Other examples of social and psychological interventions may be found in reviews by the Cochrane Collaboration (for example, the Developmental, Psychosocial, and Learning Problems Group and the Cochrane Public Health Group) and the Campbell Collaboration [8,9].

To understand their effects and to keep services up to date, academics, policymakers, journalists, clinicians, and consumers rely on research reports of intervention studies in scientific journals. Such reports should explain the methods, including the design, delivery, uptake, and context of interventions, as well as subsequent results. Accurate, complete, and transparent reporting is essential for readers to make best use of new evidence, to achieve returns on research investment, to meet ethical obligations to research participants and consumers of interventions, and to minimise waste in research. However, randomised controlled trials (RCTs) are often poorly reported within and across disciplines, including criminology [10], social work [11], education [12], psychology [13,14], and public health [15]. Biomedical researchers have developed guidelines to improve the reporting of RCTs of health-related interventions [16]. However, many social and behavioural scientists have not fully adopted these guidelines, which may not be wholly adequate for social and psychological interventions in their current form [10,13,17,18]. Because of the unique features of these interventions, updated reporting guidance is needed.

This article describes the development of a reporting guideline that aims to improve the quality of reports of RCTs of social and psychological interventions. We explain how reporting guidelines have improved the quality of reports in medicine, and why guidelines have not yet improved the quality of reports in other disciplines. We then introduce a plan to develop a new reporting guideline for RCTs, namely, the Consolidated Standards of Reporting Trials (CONSORT)-SPI (an extension for social and psychological interventions), which will be written using recommend techniques for guideline development and dissemination [19]. Wide stakeholder involvement and consensus are needed to create a useful, acceptable, and evidence-based guideline, so we hope to recruit stakeholders from multiple disciplines and professions.

A randomised trial is not the only rigorous method for evaluating interventions; many alternatives exist when RCTs are not possible or appropriate due to scientific, practical, and ethical concerns [20]. Nonetheless, RCTs are important to policymakers, practitioners, scientists, and service users, as they are generally considered the most valid and reliable research method for estimating the effectiveness of interventions [21]. Moreover, many of the issues faced in reporting RCTs also relate to other evaluation designs. As a result, this project will focus on standards for RCTs, which could then also inform the development of future guidelines for other evaluation designs.

### Impact of CONSORT guidelines

Reporting guidelines list (in the form of a checklist) the minimum information required to understand the methods and results of studies. They do not prescribe research conduct, but facilitate the writing of transparent reports by authors and appraisal of reports by research consumers. For example, the Consolidated Standards of Reporting Trials (CONSORT) Statement 2010 is an evidence-based guideline; to identify items, the developers reviewed evidence of trial design and conduct that could contribute to bias. Using consensus methods, they developed a checklist of 25 items and a flow diagram [16]. CONSORT has improved the reporting of thousands of medical experiments [22]. It has been endorsed by over 600 journals [23], and it is supported by the Institute of Educational Sciences [12]. CONSORT is the only guideline for reporting RCTs that has been developed with such rigour, and it has remained more prominent than any other guideline for over 15 years; for greatest impact, then, any further reporting guidelines related to RCTs should be developed in collaboration with the CONSORT Group.

### Limitations of previous reporting guidelines for social and psychological interventions

Researchers and journal editors in the social and behavioural sciences are generally aware of CONSORT but often object that it is not fully appropriate for social and psychological interventions [10,13,17,18]. As a result, uptake of CONSORT guidelines in these disciplines is low. While some criticisms are due to inaccurate perceptions about common features of RCTs across disciplines, many relate to real limitations for social and psychological interventions [24]. For example, CONSORT is most relevant to RCTs in medical disciplines; it was developed by biostatisticians and medical researchers with minimal input from experts in other disciplines. Journal editors, as well as social and behavioural science researchers, believe there is a need to include appropriate stakeholders in developing a new, targeted guideline to improve uptake in their disciplines [12,25]. The CONSORT Group has produced extensions of the original CONSORT Statement relevant to social and psychological interventions, such as additional checklists for cluster [26], non-pharmacological [27], pragmatic [28], and quality-of-life RCTs [29]. These extensions provide important insights, but complex social and psychological interventions, for example, include multiple, interacting components at several levels with various outcomes. These RCTs require use of several extensions at once, creating a barrier to guideline uptake; increasing intervention complexity also gives rise to new issues that are not included in existing guidelines. Therefore, simply disseminating CONSORT guidelines as they stand is insufficient, as this would not address the need for editors and authors to buy into this process. To improve uptake in these disciplines, CONSORT guidelines need to be extended to specifically address the important features of social and psychological interventions.

Social and behavioural scientists have developed other reporting guidelines, including the Workgroup for Intervention Development and Evaluation Research (WIDER) Recommendations for behavioural change interventions [14,30], the American Educational Research Association’s (AERA) Standards for Reporting Research [31], the Reporting of Primary Empirical Research Studies (REPOSE) guidelines for primary research in education [32], and the Journal Article Reporting Standards (JARS) of the American Psychological Association (APA) [33]. Whilst they address issues not covered by the CONSORT Statement and its extensions, these guidelines (except for JARS [33]) do not provide specific guidance for RCTs. Moreover, compared with the CONSORT Statement and its official extensions, guidelines in the social and behavioural sciences have not consistently followed optimal techniques for guideline development and dissemination that are recommended by international leaders in the advancement of reporting guidelines [19], such as the use of systematic literature reviews and formal consensus methods to select reporting standards [34]. Researchers in public health, psychology, education, social work, and criminology have noted that these guidelines could be more ‘user-friendly’ , and dissemination could benefit from up-to-date knowledge transfer techniques [11-13,17,30,35,36].

For example, JARS - a notable and valuable guideline for empirical psychological research - is endorsed by few journals outside of the APA, whereas CONSORT is endorsed by hundreds of journals internationally. According to ISI Web of Knowledge and Google Scholar citations, JARS is cited approximately a dozen times annually, while CONSORT guidelines are cited hundreds of times per year. Moreover, the APA commissioned a select group of APA journal editors and reviewers to develop JARS, and the group based most of their work on existent CONSORT guidelines; by comparison, official CONSORT extensions have been developed using rigorous consensus methods, have involved various international stakeholders in guideline development and dissemination, and updated content on the most recent scientific literature. Nonetheless, no current CONSORT guideline adequately addresses the unique features of social and psychological interventions. This new CONSORT extension will incorporate lessons from previous extensions, reporting guidelines, and the research literature to aid the critical appraisal, replication, and uptake of this research.

## Discussion

### Aspects of internal validity

Internal validity is the extent to which the results of a study may be influenced by bias. Like other study designs, the validity of RCTs depends on high-quality execution. Poorly conducted RCTs can produce more biased results than well-conducted RCTs and well-conducted non-randomised studies [37,38]. For example, evidence indicates that RCTs that do not adequately conceal the randomisation sequence can exaggerate effect estimates by up to 30% [39], while low-quality reports of these RCTs are associated with effect estimates exaggerated by up to 35% [40]. Social and psychological intervention RCTs are susceptible to these risks of bias as well.

Some aspects of internal validity, although included in CONSORT, remain poorly reported, even in the least complex social and psychological intervention studies. Reports of RCTs should describe procedures for minimising selection bias, but they often omit information about random sequence generation and allocation concealment [35,41], and psychological journals report methods of sequence generation less frequently than medical journals [13]. A review of educational reports found no studies that adequately reported allocation concealment [12], and reports in criminology often lack information about randomisation procedures [10,25]. RCTs of social and psychological interventions may also use non-traditional randomisation techniques, such as stepped-wedge or natural allocation [42], which need to be thoroughly described. In addition, reports of social and psychological intervention trials often fail to include details about trial registration, protocols, and adverse events [35,41], which may include important negative consequences at individual, familial, and community levels.

Other aspects of CONSORT may require greater emphasis or modification for RCTs of social and psychological interventions. In developing this CONSORT extension, we expect to identify new items and to adapt existing items that relate to the internal validity. These may include items discussed during the development of previous CONSORT extensions or other guidelines, as well as items suggested by participants in this project. For example, it may not be possible to blind participants and providers of interventions, but blinding of outcome assessors is often possible but rarely reported, and few studies explain whether blinding was maintained or how lack of blinding was handled [17,35,41]. In social and psychological intervention studies, outcome measures are often subjective, variables may relate to latent constructs, and information may come from multiple sources (for example, participants or providers). While it is an issue in other areas of research, the influence on RCT results of the quality of subjective outcome measures in social and psychological intervention research has long been highlighted, given their prevalence in social and psychological intervention research [43]. Descriptions of the validity, reliability, and psychometric properties of such measures are therefore particularly useful for social and psychological intervention trials, especially when they are not widely available or discussed in the research literature [26,44]. Moreover, multiple measures may be analysed in several ways, so authors need to transparently report which procedures were performed and to explain their rationale.

### Aspects of external validity

External validity is the extent to which study results are applicable in other settings or populations. Currently, given that RCTs are primarily designed to increase the internal validity of study findings, the CONSORT Statement gives relatively little attention to external validity. While high internal validity is an important precondition for any discussion of an RCT’s external validity, updating the CONSORT Statement to include more information about external validity is critical for the relevance and uptake of a CONSORT extension for social and psychological interventions. These interventions may be influenced by context, as different underlying social, institutional, psychological, and physical structures may yield different causal and probabilistic relations between interventions and observed outcomes. Contextual information is necessary to compare the effectiveness of interventions across time and place [45]. Lack of information relevant to external validity may prevent practitioners or policymakers from using evidence appropriately to inform decision making, yet existing guidelines do not adequately explain how authors should describe (a) how interventions work, (b) for whom, and (c) under what conditions [46].

First, it is useful for authors to explain the key components of interventions, how those components could be delivered, and how they relate to the outcomes selected. At present, authors can follow current standards for reporting interventions without providing adequate details about complex interventions [47]. Many reports neither contain sufficient information about the interventions tested nor reference treatment manuals [48]. Providing logic models - as described in the UK Medical Research Council (MRC) Framework for Complex Interventions [49] - or presenting theories of change can help elucidate links in causal chains that can be tested, identify important mediators and moderators, and facilitate syntheses in reviews [50]. Moreover, interventions are rarely implemented exactly as designed, and complex interventions may be designed to be implemented with some flexibility, in order to accommodate differences across participants [4], so it is important to report how interventions were actually delivered by providers and actually received by participants [51]. Particularly for social and psychological interventions, the integrity of implementing the intended functions and processes of the intervention are essential to understand [4]. As RCTs of a particular intervention can yield different relative effects depending on the nature of the control groups, information about delivery and uptake should be provided for all trial arms [52].

Second, reports should describe recruitment processes and representativeness of samples. Participants in RCTs of social and psychological intervention are often recruited outside of routine practice settings via processes that differ from routine services [31]. An intervention that works for one group of people may not work for people living in different cultures or physical spaces, or it may not work for people with slightly different problems and co-morbidities. Enrolling in an RCT can be a complex process that affects the measured and unmeasured characteristics of participants, and recruitment may differ from how users normally access interventions. Well-described RCT reports will include the characteristics of all participants (volunteers, those who enrolled, and those who completed) in sufficient detail for readers to assess the comparability of the study sample to populations and in everyday services [31,33,53].

Finally, given that these interventions often occur in social environments, reports should describe factors of the RCT context that are believed to support, attenuate, or frustrate observed effects [54]. Interventions may differ across groups of different social or socioeconomic positions, and equity considerations should be addressed explicitly [55,56]. Several aspects of setting and implementation may be important to consider, such as administrative support, staff training and supervision, organisational resources, the wider service system, and concurrent political or social events [5,47,57,58]. Reporting process evaluations may help understand mechanisms and outcomes.

### Developing a new CONSORT extension

This new reporting guideline for RCTs of social and psychological interventions will be an official extension of the CONSORT Statement. Optimally, it will help improve the reporting of these studies. Like other official CONSORT extensions [26,28,59,60], this guideline will be integrated with the CONSORT Statement and previous extensions, and updates of the CONSORT Statement may incorporate references to this extension.

The project is being led by an international collaboration of researchers, methodologists, guideline developers, funders, service providers, journal editors, and consumer advocacy groups. We will be recruiting participants in a manner similar to other reporting guideline initiatives - identifying stakeholders through literature reviews, the International Advisory Group for the project, and stakeholder-initiated interest in the project [14,16]. We hope to recruit stakeholders with expertise from all related disciplines. To enlist participants from all regions of the world, including low- and middle-income countries, there will be opportunities for online participation in guideline development, and we have also secured financial support for participants attending the face-to-face consensus meeting. Methodologists will identify items that relate to known sources of bias, and they will identify items that facilitate systematic reviews and research synthesis. Funders will consider how the guideline can aid the assessment of grant applications for RCTs and methodological innovations in intervention evaluation. Practitioners will identify information that can aid decision making. Journal editors will identify practical steps to implement the guideline and to ensure uptake.

We will use consensus techniques to reduce bias in group decision making and to promote widespread guideline uptake and knowledge translation activities upon project completion [61]. Following rigorous reviews of existing guidelines and current reporting quality, we will conduct an online Delphi process to identify a prioritised list of reporting items to consider for the extension. That is, we will invite a group of experts to electronically answer questions about reporting items and to suggest further questions. We will circulate their feedback to the group and ask a second round of questions. The Delphi process will capture a variety of international perspectives and allow participants to share their views anonymously. Following the Delphi process, we will host a consensus meeting to review the findings and to generate a list of minimal reporting standards, mirroring the development of previous CONSORT guidelines [16,27,28].

Together, participants in this process will create a checklist of reporting items and a flowchart for reporting social and psychological intervention RCTs. In addition, we will develop an Explanation and Elaboration (E&E) document to explain the scientific rationale for each recommendation and to provide examples of clear reporting; a similar document was developed by the CONSORT group to help disseminate a better understanding for each included checklist item [62]. This document will help persuade editors, authors, and funders of the importance of the guideline. It will be a useful pedagogical tool, helping students and researchers understand the methods for conducting RCTs of social and psychological interventions, and it will help authors meet the guideline requirements [19].

The success of this project depends on widespread involvement and agreement among key international stakeholders in research, policy, and practice. For example, previous developers have obtained guideline endorsement by journal editors who require authors and peer reviewers to use the guideline during manuscript submission, and who must enforce journal article word limits [19,63]. Many journal editors have already agreed to participate, and we hope other researchers and stakeholders will volunteer their time and expertise.

## Conclusion

Reporting guidelines help us use scarce resources efficiently and ethically. Randomised controlled trials are expensive, and the public have a right to expect returns on their investments through transparent, usable reports. When RCT reports cannot be used (for whatever reason), resources are wasted. Participants contribute their time and put themselves at risk of harm to generate evidence that will help others, and researchers should disseminate that information effectively [17]. Policymakers benefit from research when developing effective, affordable standards of practice and choosing which programmes and services to fund. Administrators and managers are required to make contextually appropriate decisions. Transparent reporting of primary studies is essential for their inclusion in systematic reviews that inform these activities. For example, there is the need to determine if primary studies are comparable, examine biases within included studies, assess the generalisability of results, and implement effective interventions. Finally, we hope this guideline will reduce the effort and time required for authors to write reports of RCTs.

A randomised controlled trial is not the only valid method for evaluating interventions [20], nor is it the only type of research that would benefit from better reporting [64]. Colleagues have identified the importance of reporting standards for other types of research, including observational [65], quasi-experimental [66], and qualitative studies [67]. This guideline is the first step towards improving reports of many designs for evaluating social and psychological interventions, which we hope will be addressed by this and future projects. We invite stakeholders from disciplines that frequently research these interventions to join this important effort and participate in guideline development by visiting our website, where they can find more information about the project, updates on its progress, and sign up to be involved (http://tinyurl.com/CONSORT-study).

## Abbreviations

AERA: American Educational Research Association; APA: American Psychological Association; CONSORT: Consolidated Standards of Reporting Trials; E&E: Explanation and elaboration; JARS: Journal Article Reporting Standards; MRC: Medical Research Council; MST: Multisystemic therapy; RCT: Randomised controlled trial; REPOSE: Reporting of Primary Empirical Research Studies; SPI: Social and psychological interventions; WIDER: Workgroup for Intervention Development and Evaluation Research.

## Competing interests

The authors declare they have no competing interests.

## Authors’ contributions

PM, EMW, and SG conceived the idea for the project. PM, EMW, SG, SH, GM, and DM helped to draft the manuscript, and have read and approved the final manuscript.
